# SG-APSIC1027: COVID-19 vaccination strategy in Singapore—Perspectives and lessons from primary care

**DOI:** 10.1017/ash.2023.26

**Published:** 2023-03-16

**Authors:** Sky Wei Chee Koh, Victor Loh Weng Keong, Liow Yiyang, Leong Choon Kit, Doris Young

**Affiliations:** National University Polyclinics, Singapore; Yong Loo Lin School of Medicine, Singapore; National University Polyclinics, Singapore; Yong Loo Lin School of Medicine, Singapore; Yong Loo Lin School of Medicine, Singapore

## Abstract

**Objectives:** The disruptions wrought by COVID-19 have spurred the development of vaccines at a pace unprecedented in global history. We have witnessed vaccine development from in vivo testing to population-wide implementation in just under 1 year. Singapore’s vaccination rate of 80%, attained at the start of September 2021, marks a milestone. It signals that plans to shift from a “zero transmission” approach to an endemic “living with COVID-19” approach is headed in the right direction, albeit cautiously and with some uncertainty. Although we ask ourselves at what rate our society should be reopened, we acknowledge that such questions are not easily answered because newer variants are proving more transmissible and, possibly, vaccine resistant compared to earlier variants. **Methods:** COVID-19 vaccination milestones were plotted. A timeline was used to map key events of Singapore’s vaccination strategy in terms of legislation, logistics and operations, vaccination eligibility, vaccination sites, and measures implemented to encourage vaccine uptake. These factors were compared with Singapore’s vaccination rate from December 2020 to early September 2021. **Results:** The successful vaccination strategy in Singapore has been explored in 4 main areas: good leadership and evidence-based decision making, use of communications, utilizing existing logistics, and an ever-ready primary care. **Conclusions:** As we transition to our second year of combating COVID-19, emerging variants, spread despite vaccination, and the contesting voices of antivaxxers pose new challenges. Vaccine-generated immunity is only one, albeit an important, element of a comprehensive COVID-19 strategy. The strategy must also entail surveillance, self-testing, contact tracing, quarantine, legislation, financial support, and strengthened social responsibility. As providers of vaccination and translators of upstream evidence and policy decisions in the community, primary care providers should be involved early in decision making regarding interventions in the community because they can foresee challenges on the ground. Let us put our continued trust in primary care providers to contribute to making Singapore a COVID-19–resilient nation.

